# The effects of the photoperiod-insensitive alleles, *se13*, *hd1* and *ghd7*, on yield components in rice

**DOI:** 10.1007/s11032-013-9994-x

**Published:** 2013-11-30

**Authors:** Quan Xu, Hiroki Saito, Ikuo Hirose, Keisuke Katsura, Yoshihiro Yoshitake, Takayuki Yokoo, Takuji Tsukiyama, Masayoshi Teraishi, Takatoshi Tanisaka, Yutaka Okumoto

**Affiliations:** Graduate School of Agriculture, Kyoto University, Kitashirakawa-Oiwakecho, Sakyo-ku, Kyoto, 606-8502 Japan

**Keywords:** Flowering time, Grain yield, Photoperiod-insensitive allele, Pleiotropic effect, Rice

## Abstract

Flowering time is closely associated with grain yield in rice (*Oryza sativa* L.). In temperate regions, seasonal changes in day length (known as the photoperiod) are an important environmental cue for floral initiation. The timing of flowering is important not only for successful reproduction, but also for determining the ideal balance between vegetative growth and reproductive growth duration. Recent molecular genetics studies have revealed key flowering time genes responsible for photoperiod sensitivity. In this study, we investigated the effect of three recessive photoperiod-insensitive alleles, *se13*, *hd1* and *ghd7*, on yield components in rice under *Ehd1*-deficient genetic background conditions to ensure vegetative growth of each line. We found that *se13*-bearing plants had fewer panicles, *hd1*-bearing plants showed decreased grain-filling percentage, and *ghd7*-bearing plants appeared to have fewer grains per panicle and fewer secondary branches. Our results indicate that the pleiotropic effects of photoperiod-insensitive genes on yield components are independent of short vegetative growth. This will provide critical information which can be used to create photoperiod-insensitive varieties that can be adapted to a wide range of latitudes.

## Introduction

Flowering time is closely associated with grain yield in rice (*Oryza sativa* L.). The seasonal change in day length (known as the photoperiod) is a key environmental cue to initiate floral induction (Hayama and Coupland 2004; Yano et al. 2001). The timing of flowering is an important agricultural trait not only for successful reproduction, but also for the appropriate balance between vegetative growth and reproductive growth duration. In rice, a facultative short-day plant, decreasing day length is a critical environmental signal for the phase transition from the vegetative to the reproductive phase (Itoh et al. 2010). In temperate regions, where seasonal changes in day length are distinct, most of the rice varieties exhibit appropriate photoperiod sensitivity to maximize their productivity. Therefore, photoperiodic sensitivity is a critical agronomic trait that can be exploited to improve the adaptability of rice varieties to a wide range of latitudes, altitudes and seasons (Izawa 2007).

Recent studies have revealed details of the genetic regulation of photoperiod sensitivity (PS). In rice, PS is determined by the antagonistic regulation of the inhibiting pathways under long day (LD) conditions and the promoting pathways that are suppressed by the inhibiting pathways (Komiya et al. 2009). Among the inhibiting pathways, *Hd1* inhibits flowering by suppressing the expression of *Hd3a* which encodes the flowering signal called florigen (Kojima et al. 2002; Yano et al. 2000). *Se13* encodes a key enzyme for the biosynthesis of the phytochrome chromophore that is indispensable for receiving the day-length signal via red and/or far-red light (Saito et al. 2011). *Ghd7* inhibits flowering under LD by suppressing the expression of *Ehd1* which is an activator of the florigen-like genes, *Hd3a* and *RFT1* (Xue et al. 2008; Doi et al. 2004; Komiya et al. 2008). *Hd1* and *Ghd7*, which are known to be core PS genes, independently control PS and the pleiotropic effects on yield-related traits of rice (Tsuji et al. 2011). The combined effects of *Hd1* and *Ehd1* reduce the number of primary branches in panicles (Endo-Higashi and Izawa 2011). Transgenic plants bearing *Hd3a* showed changes in multiple traits of vegetative organs, such as elongation of internodes and increased number of tillers (Tamaki et al. 2007). Finally, *Ghd7* affects the grain number (Xue et al. 2008). However, the genetic effects of these genes have not been investigated against the same genetic background to compare their effects on yield components.

Photoperiod insensitivity is an important trait that can be exploited to breed a valuable rice variety adapted to a wide range of latitudes (Khush 2001). Up to now, several kinds of loss-of-function alleles at the PS gene loci have been used to make rice photoperiod-insensitive. However, the effects of photoperiod-insensitive alleles in yield traits should be clarified in order to utilize them for breeding a valuable photoperiod-insensitive variety. In temperate regions, however, photoperiod-insensitive lines flower too early to achieve sufficiently long vegetative growth periods. Their excessively short vegetative growth periods are accompanied by the abnormal development of their reproductive organs such as the panicle and inflorescence. Thus, it is difficult to distinguish the exact effects of photoperiod-insensitive alleles on yield components from their indirect effects caused by the shortened vegetative growth period.

To overcome this obstacle, we evaluated photoperiod-insensitive alleles at the PS locus with the combined use of an *ehd1* allele, which is a recessive non-functional allele of *Ehd1*. The *ehd1* allele lacks the flower-promoting effect, which ensures sufficient vegetative growth. In this study, we investigated the effects of three recessive photoperiod-insensitive alleles, *se13*, *hd1* and *ghd7*, on yield components by combining them with *ehd1*. We found that these three alleles have distinctly different effects on yield components.

## Materials and methods

### Plant materials

We used three heading-time mutants, X61, HS110 and Hs169, which originated from the same *japonica* variety known as Gimbozu (GB) (Table 1). X61 harbors a recessive non-functional allele at the *Se13* locus and completely lacks a photoperiodic response (Saito et al. 2011). HS110 harbors a photoperiod-insensitive allele at the *Hd1* locus (Yano et al. 2000). HS169 is a late heading-time mutant with a recessive non-functional allele at the *Ehd1* locus (Saito et al. 2009). In addition to the three mutant lines of GB, EG2 was also used for this study. EG2 is a tester line of heading-time genes derived from a cross between Aikoku and GB. Aikoku is a landrace that harbors a recessive photoperiod-insensitive allele at the *E1* locus, which is identical to the *Ghd7* locus (Yamagata et al. 1986). GB was developed from Aikoku by a natural mutation. Thus, the four lines in this study, X61, HS110, HS169 and EG2, share the same genetic background as GB. To create the double recessive line (DMG), we performed the three cross combinations of X61 × HS169, HS110 × HS169 and EG2 × HS169 to create DMG2, DMG3 and DMG10, respectively.

**Table 1:** Genotypes, days to flowering and photoperiod sensitivity under the three photoperiod conditions of the lines used in this study

Line	Genotype				Days to heading^a^			Photoperiod sensitivity (PS)^b^
	*Hd1*	*Se13*	*Ghd7*	*Ehd1*	SD	LD	ND	
Gimbozu	*Hd1*	*Se13*	*Ghd7*	*Ehd1*	59.8	91.8	94.0	32.0
X61	*Hd1*	*se13*	*Ghd7*	*Ehd1*	61.5	65.9	60.2	4.5
HS110	*hd1*	*Se13*	*Ghd7*	*Ehd1*	61.4	75.5	76.0	14.1
EG2	*Hd1*	*Se13*	*ghd7*	*Ehd1*	55.2	79.5	77.8	24.3
HS169	*Hd1*	*Se13*	*Ghd7*	*ehd1*	74.2	97.2	101.1	23.0
DMG2	*Hd1*	*se13*	*Ghd7*	*ehd1*	84.2	89.0	85.2	4.8
DMG3	*hd1*	*Se13*	*Ghd7*	*ehd1*	79.9	88.1	84.1	8.2
DMG10	*Hd1*	*Se13*	*ghd7*	*ehd1*	75.3	94.5	90.0	19.2

^a^Days to heading from sowing under short-day (10-h light: *SD*), long-day (14.5-h light: *LD*) and natural-day-length (*ND*) conditions

^b^Photoperiod sensitivity (PS) is calculated as the difference in days to heading between SD and LD conditions

### Field experiments

All the materials were grown in the paddy rice field at the Experimental Farm of Kyoto University (Osaka, Japan; 34°51′N, 135°37′E) during the summer of 2012. Seeds were sown in the seedling nursery on 24 April and transplanted with one seedling per hill on 23 May. Each plot was 5.4 m^2^ and included 120 plants with planting densities (hill per m^2^) of 22.2 (30 cm × 15 cm intervals). Plots were arranged in a randomized block design with three replications. Fertilizers were applied with a basal dressing amount of 60 kg N per hectare, 90 kg P per hectare and 90 kg K per hectare.

### Measurements of yield components

To measure the growth rate of the plants, leaf emergence was periodically observed on the main shoot in three plants per plot. The number of tillers of three plants per plot was also counted weekly. At the full heading stage, when 90 % of the stems of each plant had emerged from the panicle, the above-ground parts of eight plants were measured from each plot. These samples were then harvested and dried in an oven at 80 °C for 2 days to measure their dry weight. Next, at the mature stage (35 days after the full heading stage), the above-ground parts of 24 plants were harvested from each plot. After counting the panicle number of these plants, the panicles were hand-threshed and put into water. The filled grains, which sank in water, were separated from unfilled grains, which did not sink in water. The unfilled grains were further classified into empty (non-fertilized grains) and partially filled grains (poorly developed grains) with the naked eye. Seed sterility was determined by the ratio of non-fertilized grains to total grains. The filled and unfilled grains were then oven-dried at 80 °C for 2 days to measure their dry weight. The number of grains per panicle and grain-filling percentage were calculated based on the above data. Three average-sized panicles were taken from each plot to observe the number of primary branches, number of secondary branches, and number of spikelets on each branch. In addition to these morphological traits, the fraction of radiation interception (FRI) was measured by a linear photosynthetically active radiation (PAR) ceptometer (AccuPAR, Decagon, USA) under a cloudy sky. The measurement of FRI was conducted from the transplant stage until the mature stage at 1-week intervals. The daily FRI was interpolated by two successive measurements. The daily intercepted radiation was calculated using the incident photosynthetically active radiation measured by the weather station located at the edge of the field.

## Results

Under normal day-length conditions (ND), X61 (*se13/Ehd1*), HS110 (*hd1/Ehd1*) and EG2 (*ghd7/Ehd1*) headed (= flowered) earlier than GB, taking 33.8, 18.0 and 16.2 days, respectively. On the other hand, DMG2 (*se13/ehd1*), DMG3 (*hd1/ehd1*) and DMG10 (*ghd7/ehd1*) headed earlier than HS169 by 15.9, 17.0 and 11.1 days, respectively. The early heading effects of the three photoperiod-insensitive alleles were partly suppressed by the effects of *ehd1*, as we expected (Table 1). The degrees of PS, which was measured by the differences in days to heading between short day and long day, were 32.0, 4.5, 14.1 and 24.3 in GB, X61, HS110 and EG2, respectively. Of the three alleles, *se13* exhibited a distinctly different effect on the reduction of PS in the presence of the functional *Ehd1* allele. The degrees of PS were 23, 4.8, 8.2 and 19.2 in HS169, DMG2, DMG3 and DMG10, respectively. Thus, the effects of the photoperiod-insensitive alleles on PS tend to decrease when combined with *ehd1*.

Table 2 shows above-ground biomass, grain yield and yield components of the four lines. Compared to HS169, all DMG lines showed a decrease in their total biomass at the mature stage. The grain yields of DMG2 and DMG3 were significantly decreased in comparison to HS169. The reduction in grain yield was observed in all DMG lines. However, the effects of three photoperiod-insensitive alleles on yield components were not the same.

**Table 2:** Effects of the photoperiod-insensitive alleles *se13*, *hd1* and *ghd7* on yield components

Line	HS169	DMG2	DMG3	DMG10
Genotype	*ef1*-*h*	*ef1*-*h/se13*	*ef1*-*h/hd1*	*ef1*-*h/ghd7*
Dry weight at mature stage (g/m^2^)	1,297.1^aA^	1,000.3^bB^	1,097.9^bB^	1,151.2^bAB^
Grain yield (g/m^2^)	528.2^aA^	409.9^bB^	360.0^cB^	490.3^aA^
Number of panicles (/m^2^)	314.5^aA^	280.6^bA^	317.6^aA^	316.4^aA^
Number of grains (/m^2^)	33789^aA^	29498^bA^	33852^aA^	30578^abA^
Number of grains per panicle	107.5^aA^	105.5^aA^	106.8^aA^	97.0^aA^
Filled grains (%)	72.0^aA^	66.9^aAB^	49.6^bB^	74.3^aA^
1,000-grain weight (g)	21.7^aA^	20.7^aA^	21.4^aA^	21.5^aA^
Number of primary branches per panicle	15.2^bA^	15.9^abA^	16.1^aA^	15.5^abA^
Number of secondary branches per panicle	19.7^abA^	21.1^aA^	16.0^bcA^	14.7^cA^

Different lower-case letters indicate significance at 5 % probability level; different capital letters indicate significance at 1 % probability level by Duncan’s new multiple range method

**Fig. 1:**
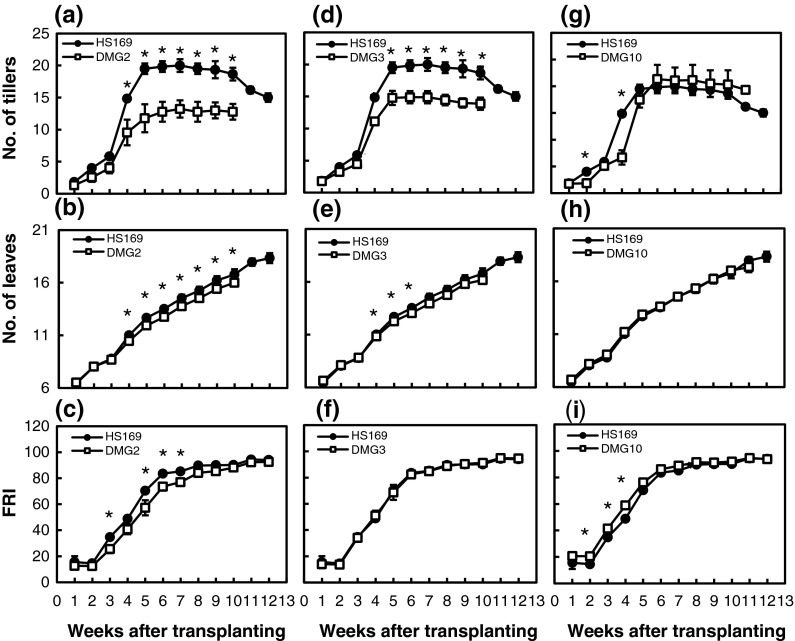
Number of tillers (**a**, **d**, **g**), leaf emergence rate (**b**, **e**, **h**) and fraction of radiation intercepted (**c**, **f**, **i**) of DMG2, DMG3 and DMG10 in comparison with HS169 during the vegetative growth period (mean ± SD). *Asterisks* indicate significant differences by Student’s *t* test at the *p* < 0.05 level

**Fig. 2:**
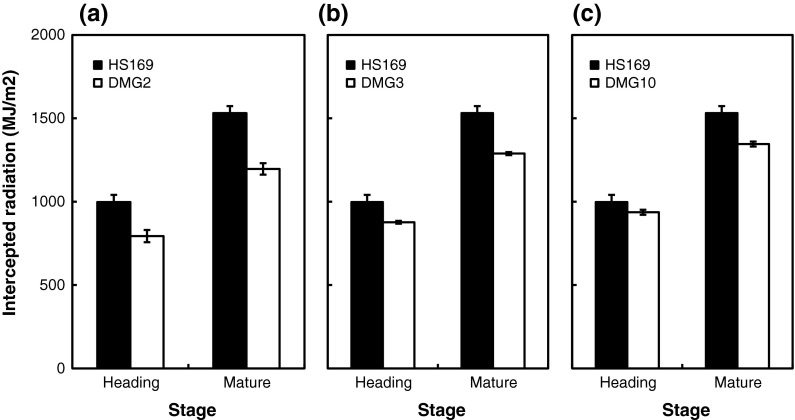
Accumulation of daily intercepted radiation at heading and mature stages of DMG2 (**a**), DMG3 (**b**) and DMG10 (**c**) in comparison with HS169

The grain yield of DMG2 was 19 % lower than that of HS169. The main cause of this reduction is the reduction in the number of grains per square meter caused by the lower number of panicles (Table 2). The grain-filling percentage and 1,000-grain weight also tended to be decreased. The number of tillers of DMG2 was fewer than that of HS169 throughout the vegetative growth period (Fig. 1a). It was apparent that the leaf emergence rates and FRI of DMG2 were lower than that of HS169 (Fig. 1b, c). The intercepted radiation was also reduced in DMG2 compared to HS169 (Fig. 2a). In DMG2, the total content of chlorophyll *a* and *b* in leaves was 4.48 mg/g, while that of HS169 was 3.62 mg/g. The deficiency of phytochromes reduced the chlorophyll content and the photosynthetic activity in HS169. This also led to a reduction in the grain-filling percentage and the 1,000-grain weight.

The grain yield of DMG3 was 28 % lower than that of HS169. This was mainly attributed to the reduction in the filled grain percentage (Table 2). Since the empty grain proportion of DMG3 was much higher than that of HS169, the low filled grain percentage of HS169 is likely caused by the failure of fertilization. Significant differences were not observed between DMG3 and HS169 in the number of panicles, the number of grains, the number of grains per panicle, or the FRI (Table 2; Fig. 1f). Although the number of tillers in DMG3 was significantly less than that of HS169 during the fifth to tenth week after transplanting, the degradation of tillers in DMG3 during the late vegetative growth period was not as obvious as that of HS169 (Fig. 1d). The number of primary branches per panicle was slightly increased, whereas the number of secondary branches tended to be decreased in DMG3. *Hd1* thus promoted the development of the secondary branches during the panicle development period.

The grain yield of DMG10 was decreased by 9 % in comparison to HS169, but this reduction was not significant (Table 2). The main cause of this reduction came from a reduction in the number of grains per square meter. The number of grains per panicle and the number of secondary branches also tended to be decreased in DMG10. In terms of panicle architecture, close observation revealed that the number of secondary branches was reduced on most of the primary branches in DMG10. The leaf emergence rate of DMG10 was almost indistinguishable from that of HS169 (Fig. 1h), whereas the number of tillers in DMG10 was less than that of HS169 during the first 4 weeks (Fig. 1g). The FRI in DMG10 was higher than that in HS169 (Fig. 1i), while the intercepted radiation in DMG10 was not different from that in HS169 (Fig. 2c).

## Discussion

Photoperiod-insensitive alleles *se13*, *hd1* and *ghd7* promote flowering and reduce biomass due to their short vegetative growth period (Tables 1, 2). Interestingly, the reduction in yield of each DMG line was caused by distinct yield components other than reduced biomass. Reduction in the grain yield of DMG2 and DMG3 was attributed to the reduction in grains per square meter and grain-filling percentage, respectively. Furthermore, in DMG2, the number of panicles per square meter, grain-filling percentage and 1,000-grain weight were also decreased. The number of secondary branches also appeared to be decreased in DMG3 and DMG10. These findings suggest that each photoperiod-insensitive allele has different effects on specific yield components.

*Se13* encodes a protein involved in the phytochromobilin biosynthesis pathway, and an *Se13*-deficient mutant failed to produce the normal amount of the holo-protein of the phytochrome. As a result, the *Se13*-deficient mutant lost its light signal responses (Saito et al. 2011). DMG2, harboring a non-functional allele at the *Se13* locus, loses the photoperiodic response and has pale green leaves due to a low content of chlorophyll *a* and *b*. Therefore, the decreased 1,000-grain weight and grain-filling percentage were caused by the low photosynthetic activity. Furthermore, the low Pfr/Pr ratio (a ratio of two types of phytochrome) enhances the apical dominant response and represses axillary shoot development (Deregibus et al. 1983; Casal et al. 1986; Smith and Whitelam 1997). Our results showed that the number of tillers in DMG2 was less than in HS169 during the entire growth period (Fig. 1a). Thus, DMG2 is not able to receive the light signal because of the deficiency of phytochromobilin and its low Pfr/Pr ratio might inhibit tiller development.

*Hd1,* which is a rice ortholog of *Arabidopsis* *CONSTANS* (CO), encodes a protein with a zinc-finger domain containing a CCT motif (Yano et al. 2000). DMG3, which harbors a non-functional photoperiod-insensitive allele due to the insertion of the transposable element *mPing* at the intron of the *Hd1* gene (Yano et al. 2000), flowered earlier than HS169 (Table 1). In DMG3, the grain-filling percentage was significantly decreased due to the failure of fertilization. Such a drastic decrease in the grain-filling percentage was not observed in other years or in its single recessive line of HS169 (*Hd1*/*ehd1*). Thus, the combined effects of *hd1* and *ehd1* cause genetic vulnerability in seed fertilization to environment factor(s). Since pollen fertility was normal in both HS169 and DMG3 (data not shown), it appears that a sufficient amount of pollen did not fall on the stigma of DMG3, possibly due to the indehiscent anther. Further research should be done to clarify the combined effects of *hd1* and *ehd1* on seed fertility. We also found that the maximum number of tillers was significantly decreased in DMG3 from the fifth week to the tenth week after transplanting, whereas the final number of tillers (= number of panicles) was not decreased (Fig. 1d). The deficiency of *hd1* potentially reduces the number of ineffective tillers in the late vegetative growth period. The details of the pleiotropic effects of *Hd1* need to be clarified much further.

*Ghd7* not only regulates heading time, but also has effects on plant height and number of grains per panicle. *Ghd7* also changes the numbers of both primary and secondary branches (Xue et al. 2008; Xing and Zhang 2010). Our results showed that the number of secondary branches was decreased in DMG10, which harbors a recessive photoperiod-insensitive allele, whereas the number of primary branches was not decreased in DGM10 (Table 2). It has also been reported that *Ehd1* reduces the number of primary branches. If *Ghd7* and *Ehd1* independently regulate the number of primary branches, the combined effects of *ghd7* and *ehd1* should be observed. However, no significant difference in the number of primary branches was observed between HS169 (*Ghd7/ehd1*) and DMG10 (*ghd7/ehd1*). This suggests that *ghd7* indirectly reduces the number of primary branches through the up-regulation of *Ehd1* expression. In addition, the number of tillers in DMG10 was less than in HS169, at least during the first 4 weeks after transplanting (Fig. 1g). Therefore, *Ghd7* might be involved in the development of axillary buds (which develop into tillers) on the main shoot and axillary buds (which develop into secondary branches) on the primary branches. DMG10 has a higher FRI than HS169, but similar intercepted radiation to HS169. This could be explained by two possible causes: the leaf area of DMG10 is larger than that of HS169, and/or DMG10 efficiently accepts solar radiation due to a less overlapping spatial arrangement of leaves. Of the three photoperiod-insensitive alleles, only *ghd7* reduced PS without decreasing the number of tillers (Fig. 1g; Table 2). This is the unique advantage of *ghd7* in the breeding of a photoperiod-insensitive variety.

Most of the improved varieties have been made insensitive to photoperiod by the introduction of the photoperiod-insensitivity gene, and can be grown during any season and in most tropical and subtropical countries (Khush 2001). Recently, improved varieties have had not only wide adaptability, but also tend to acquire the short vegetative growth duration. If the short-duration varieties produce the same amount of grain in fewer days than medium-duration varieties, their per-day productivity could become much higher (Khush 2001). The present study demonstrated the effect of three photoperiod-insensitive alleles on grain yield and yield component traits. Our results clarify the pleiotropic effects of flowering time genes on yield components independent of the shortened vegetative growth period. This will provide us with critical information that can be used to breed novel varieties with worldwide adaptability based on photoperiod insensitivity and to develop novel ecotypes to realize massive increases in rice yield.
